# Irresponsible Opinion

**Published:** 1885-04

**Authors:** 


### EDITORIAL.

IRRESPONSIBLE OPINION.

An editorial appeared in the columns of the *Chicago Daily Tribune*, on Sunday, March 15, 1885, which criticised the medical and surgical treatment of General Grant's case in unmeasured terms.

After referring to the alleged illiberal spirit displayed by the physicians in charge, in the exclusion of the "Cancer Specialist," recommended by Mr. Elkins, from consultation, the author hurls the usual stereotyped anathemas at medical science in general and physicians in particular. The editorial reminds us vividly of the heated newspaper discussions of the surgical treatment of President Garfield's case. Our readers will probably remember the extreme coolness with which the *New York Herald* suggested the propriety of deciding upon measures of treatment through the medium of mass-meetings.

An article in the March number of *Macmillan's Magazine* contains some excellent remarks, addressed more particularly to newspaper strategists, but applicable, with equal force, to people generally, who speak with positiveness about things of which they have no knowledge.

The remarks might be addressed to newspaper physicians and surgeons, and particularly to our contemporary of the *Tribune*, who makes use of the "familiar provincialism" aptly described by the author of the article in *Macmillan's Magazine*. The writer says:

"There is a familiar provincialism used in deprecation of such self-praise as is felt to be inevitable, 'Though I say it as shouldn't.' Might not this formula be extended to all such enforced judgments as I am contemplating? as thus, 'I am decidedly of the opinion that the attack at Tel-el-Kebir should have begun an hour earlier than it did, but seeing that I have been engaged all my life in the manufacture of small-clothes, and that although a volunteer I have never seen active service, 'I say it as shouldn't,'—Sir Garnet being so much more likely to be right than I.' "

And also:

"We cannot all be expected to emulate the sobriety of that Parisian bootmaker, a hero of fable—I am afraid—who on being asked his opinion of the respective merits of Turenne and the Grand Condé on the same stricken field, replied: 'I made the boots of both gentlemen; as far as boots go there is not a pin to choose between them; beyond that I cannot go, for it lies outside of my profession.' "

